# Supplementary material for: Assessing the effect of model specification and prior sensitivity on Bayesian tests of temporal signal
Source: PLoS Comput Biol. 2024 Nov 6;20(11):e1012371. doi: 10.1371/journal.pcbi.1012371 (PMC11573219; doi:10.1371/journal.pcbi.1012371)
Supplement: S1 Fig — The clock rate, root height, tree length, and coefficient of rate variation are shown under three priors on θ, exponential (red), gamma (blue), and log-normal (green). (PDF) [file pcbi.1012371.s001.pdf]

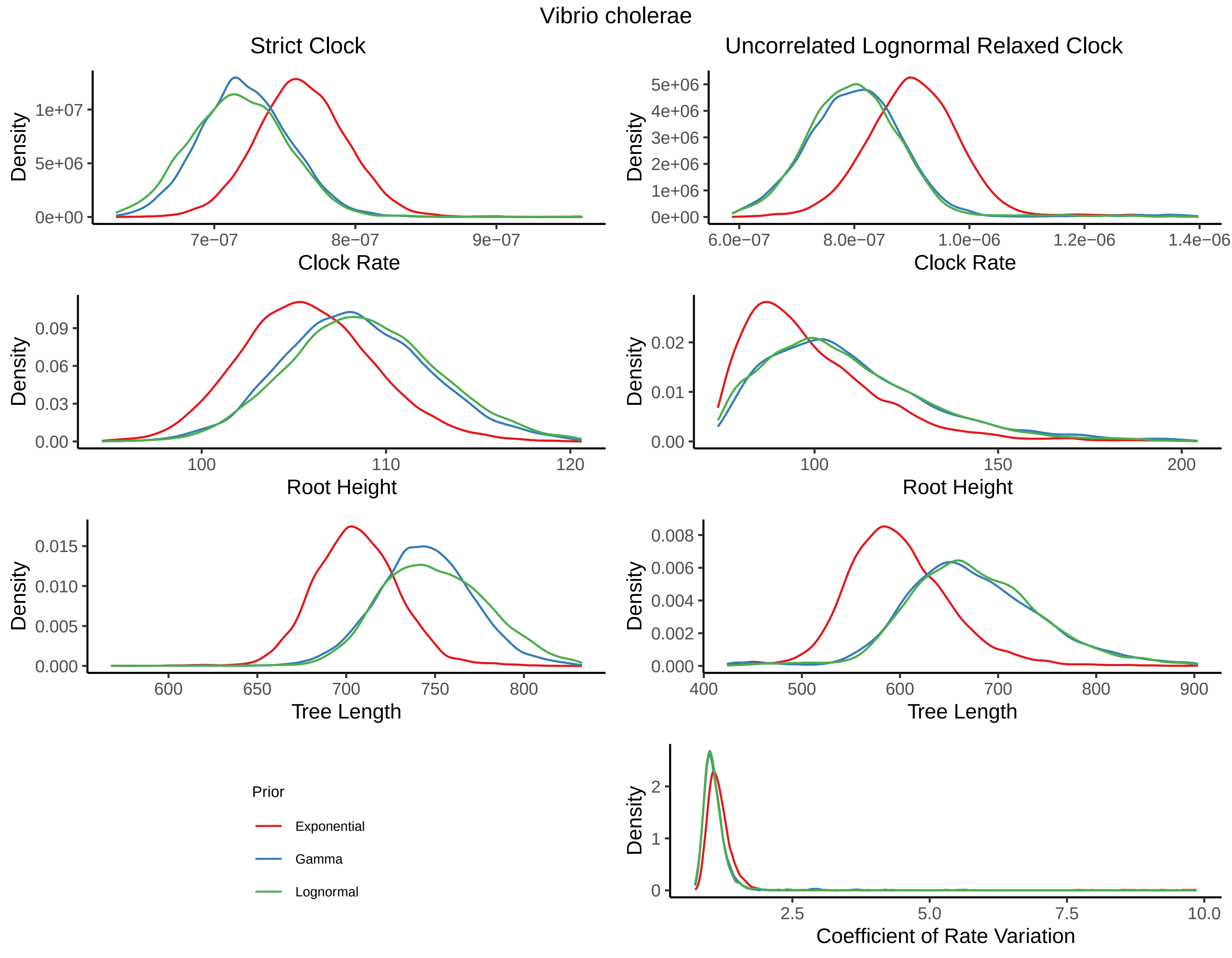
Vibrio cholerae
Strict Clock
Uncorrelated Lognormal Relaxed Clock
Density
1e+07
5e+06
0e+00
7e−07
8e−07
9e−07
Clock Rate
5e+06
4e+06
3e+06
2e+06
1e+06
0e+00
6.0e−07
8.0e−07
1.0e−06
1.2e−06
1.4e−06
Clock Rate
0.09
0.06
0.03
0.00
100
110
120
Root Height
0.02
0.01
0.00
100
150
200
Root Height
0.015
0.010
0.005
0.000
600
650
700
750
800
Tree Length
0.008
0.006
0.004
0.002
0.000
400
500
600
700
800
900
Tree Length
2
1
0
2.5
5.0
7.5
10.0
Coefficient of Rate Variation
Prior
Exponential
Gamma
Lognormal
