# Supplementary material for: Assessing the effect of model specification and prior sensitivity on Bayesian tests of temporal signal
Source: PLoS Comput Biol. 2024 Nov 6;20(11):e1012371. doi: 10.1371/journal.pcbi.1012371 (PMC11573219; doi:10.1371/journal.pcbi.1012371)
Supplement: S2 Fig — The clock rate, root height, tree length, and coefficient of rate variation are shown under three priors on θ, exponential (red), gamma (blue), and log-normal (green). (PDF) [file pcbi.1012371.s002.pdf]

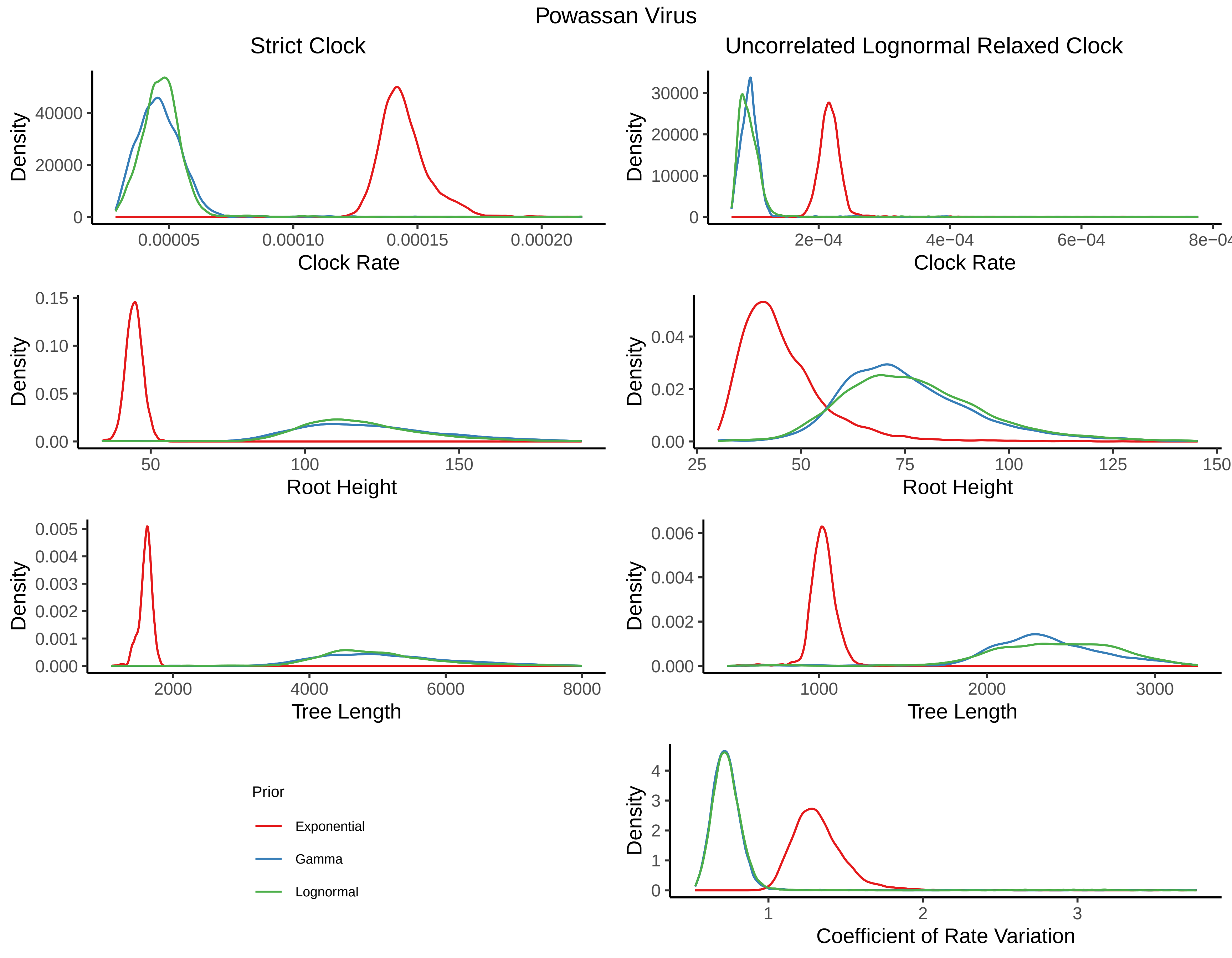

Powassan Virus
Strict Clock
Uncorrelated Lognormal Relaxed Clock
Density
Clock Rate
0.00005
0.00010
0.00015
0.00020
2e−04
4e−04
6e−04
8e−04
Root Height
Tree Length
Coefficient of Rate Variation
Prior
Exponential
Gamma
Lognormal
