# Supplementary material for: Assessing the effect of model specification and prior sensitivity on Bayesian tests of temporal signal
Source: PLoS Comput Biol. 2024 Nov 6;20(11):e1012371. doi: 10.1371/journal.pcbi.1012371 (PMC11573219; doi:10.1371/journal.pcbi.1012371)
Supplement: S4 Fig — The polygons represent the relative log marginal likelihoods of each microbe data set under a different effective population size (θ) prior, analysed with four different configurations. Het (heterochronous) includes sampling, while Iso (isochronous) does not include any sampling times. SC is strict clock and UCLD is the uncorrelated log-normal relaxed clock. Red represents an exponential prior on the effective population size, blue is a Γ prior, and green is a log-normal prior. (PDF) [file pcbi.1012371.s004.pdf]

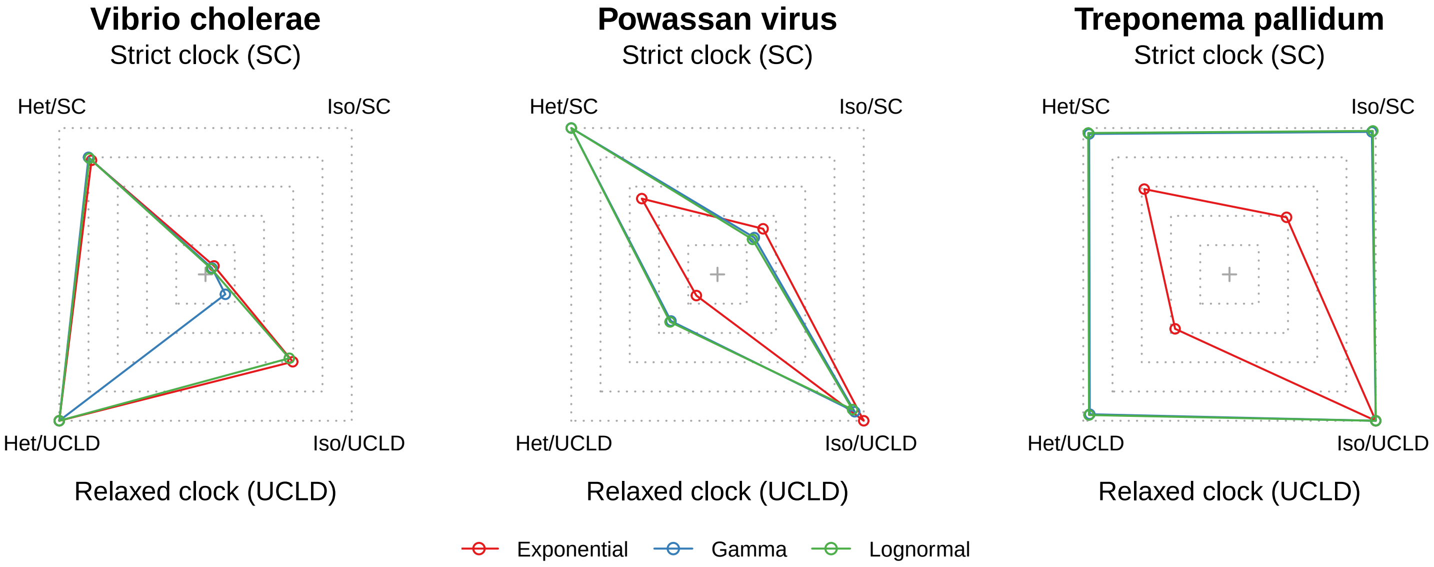
Vibrio cholerae
Strict clock (SC)
Het/SC
Iso/SC
Het/UCLD
Iso/UCLD
Relaxed clock (UCLD)
Powassan virus
Strict clock (SC)
Het/SC
Iso/SC
Het/UCLD
Iso/UCLD
Relaxed clock (UCLD)
Treponema pallidum
Strict clock (SC)
Het/SC
Iso/SC
Het/UCLD
Iso/UCLD
Relaxed clock (UCLD)
Exponential
Gamma
Lognormal
