# Supplementary material for: Assessing the effect of model specification and prior sensitivity on Bayesian tests of temporal signal
Source: PLoS Comput Biol. 2024 Nov 6;20(11):e1012371. doi: 10.1371/journal.pcbi.1012371 (PMC11573219; doi:10.1371/journal.pcbi.1012371)
Supplement: S5 Fig — The top row is for heterochronous simulations, where temporal signal is present, and the bottom row is for isochronous simulations that do not have temporal signal. Within each panel the corners correspond to a combination of model and sampling times, either a strict (SC) or relaxed molecular clock with an underlying log-normal distribution (UCLD), and with (heterochronous) or without (isochronous) sampling times. The polygons represent the relative log marginal likelihood under three possible priors on the scaled population size (Φ) parameter of the exponential-growth coalescent tree prior. The correct model used to generate the data is the SC heterochronous (SC/het) for the top row and the SC isochronous (Iso/SC) for the bottom row. Each polygon is for one simulation replicate (a total of ten) and the colours denote whether we employed a hard bound on the root height of the form Uniform(0.0, 5.0), as shown in the legend. (PDF) [file pcbi.1012371.s005.pdf]

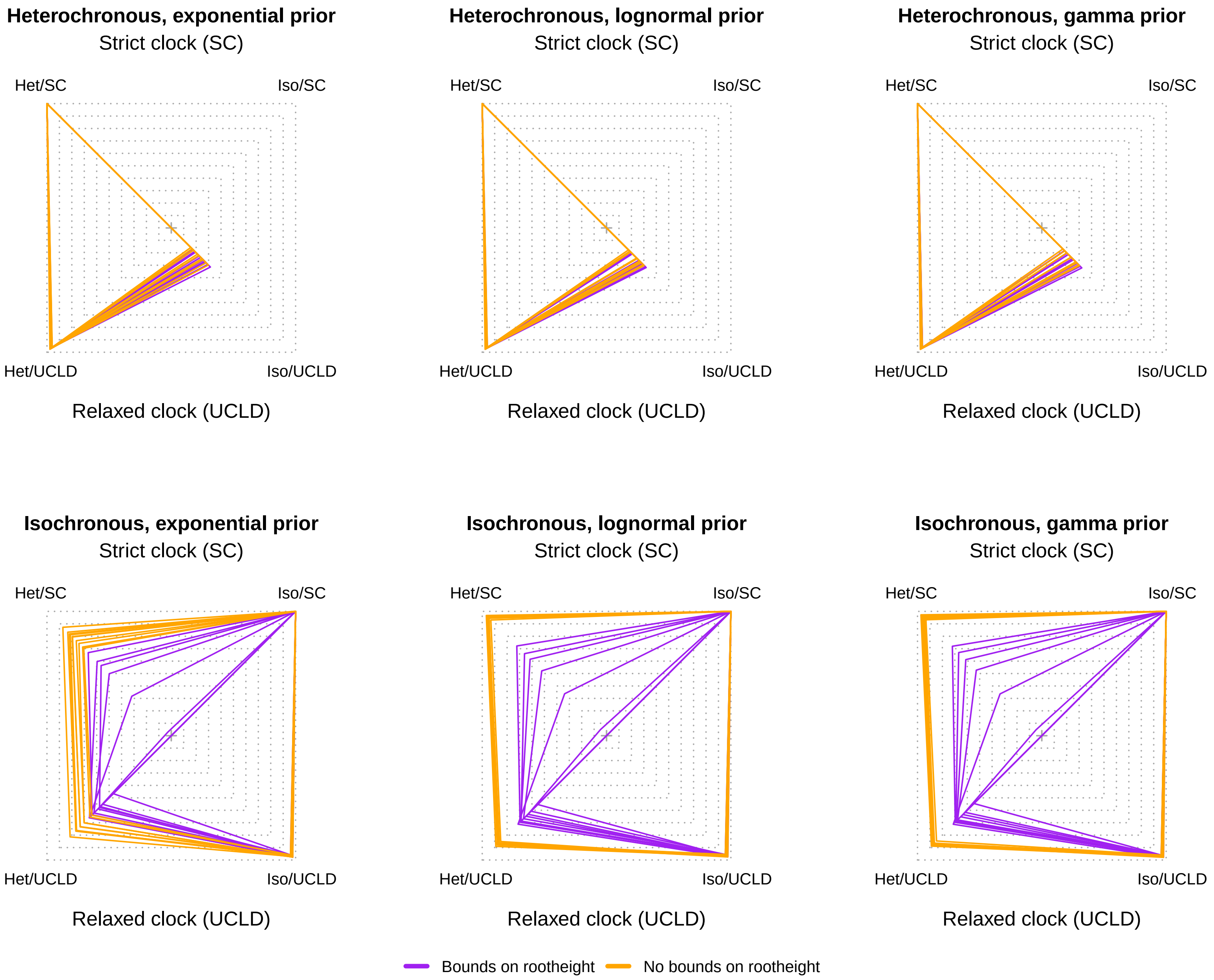
Heterochronous, exponential prior
Heterochronous, lognormal prior
Heterochronous, gamma prior
Isochronous, exponential prior
Isochronous, lognormal prior
Isochronous, gamma prior
Strict clock (SC)
Relaxed clock (UCLD)
Het/SC
Iso/SC
Het/UCLD
Iso/UCLD
Bounds on rootheight
No bounds on rootheight
