# Supplementary material for: Assessing the effect of model specification and prior sensitivity on Bayesian tests of temporal signal
Source: PLoS Comput Biol. 2024 Nov 6;20(11):e1012371. doi: 10.1371/journal.pcbi.1012371 (PMC11573219; doi:10.1371/journal.pcbi.1012371)
Supplement: S6 Fig — Phylogenetic likelihood (i.e. the probability of the sequence data, given the full model and the prior) vs the root height of phylogenetic trees. Each panel represents a data set that was truly isochronous and the prior on the effective population size of the constant-size coalescent, θ. Here we show the posterior distributions for a single data set, analysed under three different priors on θ and with sampling times that were isochronous (no sampling times), or heterochronous, and with and without hard bounds on the root height. Each point corresponds to a sample from the posterior, with shapes and colours described in the legend. Note that the isochronous (orange circles) and heterochronous without bounds have similar phylogenetic likelihoods, despite having trees with root heights that differ by multiple orders of magnitude. In practice, the heterochronous analyses without bounds produce trees with root heights that are so old that they are effectively isochronous (see Fig 6). In contrast, using hard bounds on the root height, even when the bounds are twice the true age of the root height results in a substantial penalty on the phylogenetic likelihood, and the correct classification in BETS. Note that here the exponential prior on θ results in much younger root height values than for the two other priors on this parameter. (PDF) [file pcbi.1012371.s006.pdf]

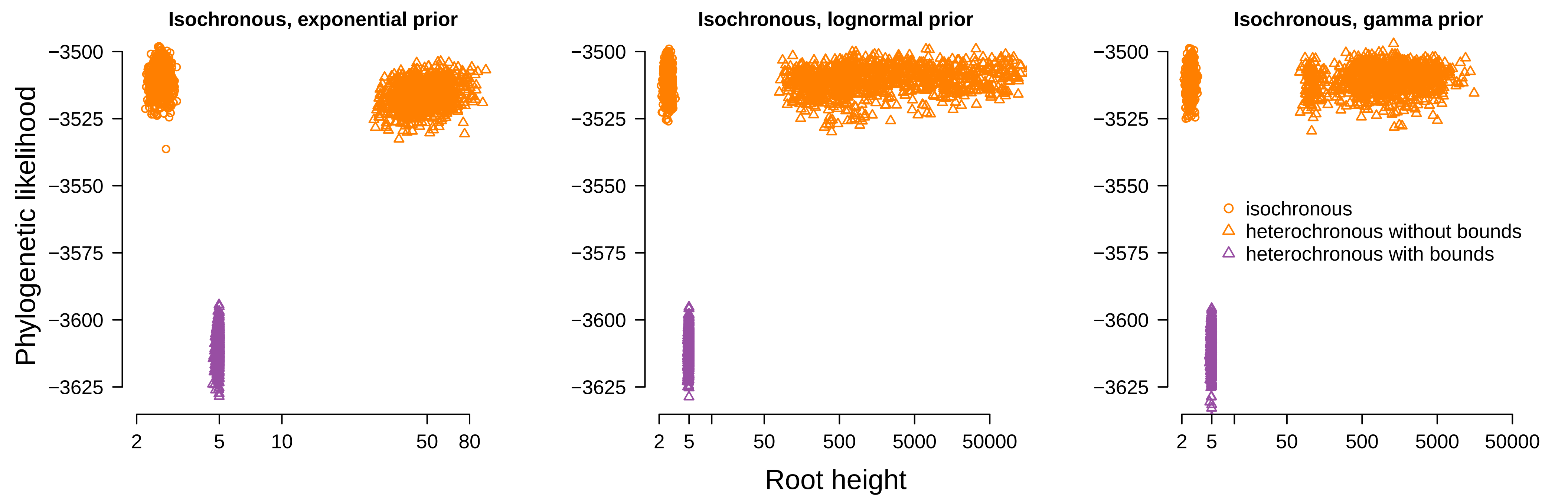
Isochronous, exponential prior
Isochronous, lognormal prior
Isochronous, gamma prior
Phylogenetic likelihood
−3500
−3525
−3550
−3575
−3600
−3625
2
5
10
50
80
500
5000
50000
Root height
isochronous
heterochronous without bounds
heterochronous with bounds
